# Large-scale self-organized gold nanostructures with bidirectional plasmon resonances for SERS†

**DOI:** 10.1039/c8ra04031a

**Published:** 2018-06-21

**Authors:** Benjamin Schreiber, Dimitra Gkogkou, Lina Dedelaite, Jochen Kerbusch, René Hübner, Evgeniya Sheremet, Dietrich R. T. Zahn, Arunas Ramanavicius, Stefan Facsko, Raul D. Rodriguez

**Affiliations:** Helmholtz-Zentrum Dresden-Rossendorf Bautzner Landstraße 400 01328 Dresden Germany; Rudolf Virchow Center, University of Würzburg Josef-Schneider-Str. 2 97080 Würzburg Germany benjamin.schreiber@uni-wuerzburg.de; Leibniz-Institut für Analytische Wissenschaften-ISAS-e.V., ISAS Berlin Schwarzschildstr. 8 12489 Berlin Germany; Vilnius University, Faculty of Chemistry and Geosciences, Department of Physical Chemistry Naugarduko 24 Vilnius Lithuania; Chemnitz University of Technology D-09107 Chemnitz Germany raulmet@gmail.com; Tomsk Polytechnic University 30 Lenin Ave 634050 Tomsk Russia

## Abstract

Efficient substrates for surface-enhanced Raman spectroscopy (SERS) are under constant development, since time-consuming and costly fabrication routines are often an issue for high-throughput spectroscopy applications. In this research, we use a two-step fabrication method to produce self-organized parallel-oriented plasmonic gold nanostructures. The fabrication routine is ready for wafer-scale production involving only low-energy ion beam irradiation and metal deposition. The optical spectroscopy features of the resulting structures show a successful bidirectional plasmonic response. The localized surface plasmon resonances (LSPRs) of each direction are independent from each other and can be tuned by the fabrication parameters. This ability to tune the LSPR characteristics allows the development of optimized plasmonic nanostructures to match different laser excitations and optical transitions for any arbitrary analyte. Moreover, in this study, we probe the polarization and wavelength dependence of such bidirectional plasmonic nanostructures by a complementary spectroscopic ellipsometry and Raman spectroscopy analysis. We observe a significant signal amplification by the SERS substrates and determine enhancement factors of over a thousand times. We also perform finite element method-based calculations of the electromagnetic enhancement for the SERS signal provided by the plasmonic nanostructures. The calculations are based on realistic models constructed using the same particle sizes and shapes experimentally determined by scanning electron microscopy. The spatial distribution of electric field enhancement shows some dispersion in the LSPR, which is a direct consequence of the semi-random distribution of hotspots. The signal enhancement is highly efficient, making our SERS substrates attractive candidates for high-throughput chemical sensing applications in which directionality, chemical stability, and large-scale fabrication are essential requirements.

## Abstract 2

Here we present a two-step fabrication of large-scale self-organized gold nanostructures for multicolor surface-enhanced Raman spectroscopy (SERS). We studied the morphology and plasmonic responses of our substrates and performed optical simulations.

## Graphical abstract

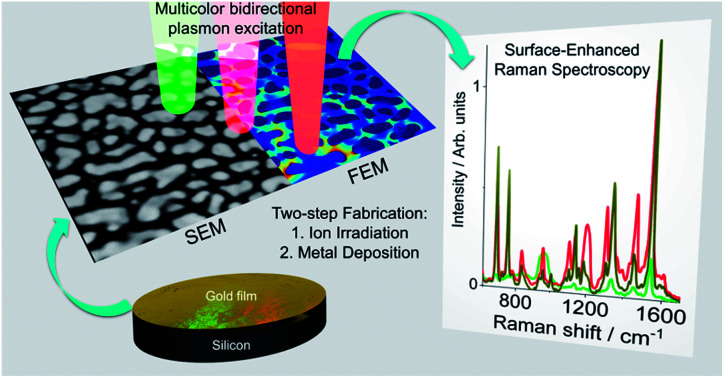

## 1 Introduction

Raman spectroscopy is a well-established method in a wide-field of chemical sensing applications.^1^ For the detection of low concentration of molecules, Raman spectroscopy (RS) reaches its limit, because of the rather low Raman scattering cross-section of most samples. The most efficient approach to overcome this problem is the application of surface-enhanced Raman spectroscopy (SERS).^2^ SERS exploits the strong electromagnetic amplifying properties of plasmonic nanostructures. Particularly interesting are plasmonic surfaces with more than one localized plasmon resonance (LSPR) that provide a broad range of applicability.^3^ With such nanostructures, it is possible to employ simultaneously complementary vibrational spectroscopy methods ranging from SERS, surface-enhanced resonant Raman spectroscopy (SERRS), and surface-enhanced infrared absorption using the same sample.^4^ However, the applicability of this kind of samples is undermined by the fabrication complexity that involves multiple steps and lithography processes.

Self-assembled SERS systems with bidirectionally excitable LSPRs offer a strategy to overcome this limitation. SERS substrates with bidirectionally excitable LSPRs supporting spectrally separated LSPRs which can be selectively triggered by polarization of the used light source. This allows broadband excitable SERS while spectral dependent Raman responses can be selectively switched on and off at the same substrate location and molecule concentration. A particularly interesting approach is the application of surface structures as a template for the self-organized alignment of molecules,^5^ colloidal nanoparticles,^6^ or vapour deposited nanoparticles.^7–9^ Template candidates are atomic terraces,^10,11^ epitaxially grown ridge-valley structures,^12,13^ and low-energy ion-induced ripple patterns on solid surfaces.^14^ These ripple structures are especially interesting, because they allow both wafer-scale fabrication on most solid surfaces and flexible ripple periodicities of several tens to hundreds of nanometers that can be controlled by the ion irradiation parameters.^15–19^ The substrates are ideal for the self-organization of plasmonic nanostructures with strong anisotropic optical properties.^20–22^ The polarization-dependent SERS response of such structures was reported for parallel gold nanowires (NWs)^23–26^ and silver nanoparticle (NP) chains.^27,28^ For gold NWs, the SERS enhancement is dominant across the ripple pattern. Along continuous metallic nanowires, a Drude-like behaviour is observed undermining the potential for surface enhancement. In contrast, noble metal nanoparticle chains on rippled templates have the potential for SERS with bidirectional independent tuneable LSPRs.^13,28–30^ It is understandable that SERS substrates that are plasmonically active under multiple excitation wavelengths have significant potential for multipurpose chemical and biochemical sensing applications. This kind of SERS structures can be produced at a large-scale with a two-step fabrication routine. Since, the SERS enhancement effect of anisotropically aligned silver nanoparticles on rippled patterns is well studied,^20,27,28^ it is time to investigate the plasmonic responses of anisotropically aligned gold nanoparticle systems for SERS applications. Gold nanostructures are known for their chemical stability,^31^ bio-compatibility, and strong plasmonic responses in red and near-infrared spectral regions^32,33^ and thus gold is a popular material choice for SERS substrates.^34^

Here we present gold nanoparticle structures that are self-organized along rippled templates. We generate these nanostructures with a two-step fabrication routine including low-energy ion beam irradiation and oblique-angle physical vapour deposition. We show that the position of the localized surface plasmon resonance (LSPR) depends on the gold nanoparticle geometry, which allows tuning the LSPRs from the visible (VIS) to the near-infrared (NIR) spectral range. The different LSPRs can be excited selectively by setting the excitation light polarization along or across the rippled structures. We study the particle growth and alignment process along the rippled template by scanning electron microscopy (SEM) and cross-sectional transmission electron microscopy (TEM). The anisotropic spectral properties are evaluated by spectroscopic ellipsometry (SE) and probed by Raman spectroscopy for three different laser lines within the VIS-NIR range. We use a 1 nm ultra-thin homogenous film of cobalt phthalocyanine (CoPc) as the Raman probe deposited simultaneously on all sample surfaces. With an ultra-homogeneous film as a SERS probe the number of molecules on a SERS substrate and reference can be directly compared. This approach allows us to make a proper evaluation of the SERS enhancement without the need of additional assumptions to estimate the number of molecules in the SERS substrates.^32^ We visualize the plasmonic hotspots by the finite element method (FEM) simulations using the exact nanoparticle size, geometry, and configuration experimentally determined from the SEM and TEM analysis.

## 2 Experimental

### 2.1 Sample fabrication

#### First step

Ripple patterns are produced by ion irradiation with a Kaufman type ion-source in a vacuum chamber. The sample was transferred into the chamber and tilted until the surface normal is oriented 67° of with respect to the ion source. Once a base pressure of ∼10^−8^ mbar was reached, argon gas was inserted into the chamber with a pressure of ∼10^−4^ mbar. Ion-beam irradiation is performed at room temperature with a broad argon ion-beam (acceleration voltage 800 V, ion flux ∼2.5 × 10^15^ cm^−2^ s^−1^). The irradiation process was finished when a total ion dose of 10^18^ cm^−2^ was reached. These process parameters led to surface ripple structures on silicon with a low defect density and a periodicity of 50 nm.^35^

#### Second Step

Oblique-angle gold deposition was performed by electron-beam deposition in a high vacuum chamber (BesTech, Germany) at ∼10^−8^ mbar working pressure and a deposition angle of 80° with respect to the surface normal and facing the long flanks of the ripple pattern. The deposition rate of ∼0.01 nm s^−1^ was controlled by a quartz micro balance (QCM). The sample thicknesses are given by the QCM recorded gold thickness. Since the QCM is calibrated for the deposition on surfaces under normal incidence angle, we checked the gold deposition with Rutherford backscattering spectrometry (RBS). For RBS, the 1.7 MeV helium ion-beam at the Helmholtz-Zentrum Dresden-Rossendorf was used. The data were analysed by the software SIMNRA.^36^ The film thicknesses of the Au nanostructures are calculated by the areal density divided by the product of surface occupation and the atomic density of gold (see ESI Fig. S1†). The average effective deposition rate for the tilted samples is (1.2 ± 0.1) pm s^−1^. The ratio of QCM and RBS measured film thicknesses are ∼cos(80°). This is in agreement with by the projected area caused by oblique deposition angle. For additional post-deposition annealing in air a conventional heating plate was used.

### 2.2 Morphological characterization

The topography of the ion-induced surface ripple pattern was measured by atomic force microscopy (AFM) in tapping mode (Bruker MM8 AFM with PPP-NCRL tips from Nanosensors). Top view images of deposited Au structures were taken by scanning electron microscopy (SEM) using a Raith e_LiNE plus with a Zeiss Gemini optics. Cross-sectional transmission electron microscopy (TEM) images were taken using an image C_s_-corrected Titan 80-300 microscope FEI ltd (Eindhoven, The Netherlands). TEM lamella preparation was performed by a Zeiss Crossbeam NVision 40 system and a Kleindiek micromanipulator. To protect the Au nanoparticles from being damaged by the focused Ga ion beam, the sample surface was initially covered by depositing a carbon-based cap layer. Prior to each TEM analysis, the specimen mounted in a double-tilt analytical holder was placed for 10 seconds into a plasma Model 1020 cleaner (Fischione) to remove contaminations.

### 2.3 Optical characterization and Raman spectroscopy

We performed spectroscopic ellipsometry (SE) with a J. A. Woollam M-2000FI device (angle of incidence = 75°, 100 repetitions per spectra) to measure the change of the phase and polarization ration of the light reflected from the sample.^37^ The plotted imaginary part of the pseudo-dielectric function, 〈*ε*_2_〉 provides the indication of light absorption.^38,39^ In our plasmonic systems, gold nanostructures are formed on a silicon surface. Light absorption above 550 nm is directly connected to excitation of surface plasmons. A peak in the 〈*ε*_2_〉 spectra indicates the LSPR position.^40^ The rotation of the sample allowed the measurement of both optical axes of sample parallel (*E⃑*_∥_) and perpendicular. (*E⃑*_⊥_) to the ripple patterns.

For Raman Spectroscopy (RS) a LabRam HR800 from Horiba Scientific equipped in back-scattering geometry was used. A HeNe Laser (632.8 nm, laser power at the sample 1 mW) was focused and the signal collected with a 50× LWD objective (numerical aperture of 0.5). The Raman signal was diffracted by a 600 lines per mm diffraction grating and recorded by an EMCCD. A 1 nm thick cobalt phthalocyanine (CoPc) film was used as a well-defined Raman probe. CoPc has an absorption maximum at 650 nm.^41,42^ Thus the molecules are resonantly excited at 632.8 nm. The samples were covered with CoPc by molecular beam evaporation in a vacuum chamber (working pressure 10^−6^ mbar). The film thickness was determined by QCM-based measurement. On SERS and reference substrates (REF) the number of CoPc molecules (*N*) was considered identical because all substrates were simultaneously covered under the same conditions of deposition (*N*_REF_ ≅ *N*_SERS_). This allows us to calculate the SERS enhancement factor directly from the Raman intensities EF_SERS_ = (*I*_SERS_/*I*_REF_)/(*N*_SERS_/*N*_REF_) ≅ *I*_SERS_/*I*_REF_. All shown Raman spectra are averages of 16 individual Raman spectra registered at different measuring points (in an 80 μm × 80 μm grid). Each single point was averaged for 10 times with 1 second acquisition time. In the ESI Fig. S4† an example of Raman signal acquisition at different locations on a sample and under different polarizations is shown. Performing SERS with an ultra-thin film of CoPc simultaneously deposited on all substrates, and with the time and spatial averages of the Raman signal it was possible to evaluate the SERS enhancement factors without minimal assumptions and statistical uncertainties.^43^

### 2.4 Finite element method calculations (FEM)

We used the wave optics module in COMSOL Multiphysics 4.4. From SEM images, we extracted the particle geometry by masking the particles greater than 5 nm (using Gwyddion^44^). The masks were contoured and saved as .dxf files (by Python 2.7 and OpenCV) to import into COMSOL. We used dielectric functions for gold,^45^ silicon,^46^ and silicon dioxide.^47^ For 2D “bird-view” calculations the gold particles were embedded in an effective medium (82.5% air and 17.5% silicon). The excitation field is implemented as an incoming wave in the model plane. For cross-section simulations we used an excitation port and continuous periodic boundary conditions. A maximum mesh size of 1 nm and 5 nm were used for the boundaries and domains, respectively. The mean field enhancement was calculated by the area mean value of |*E*/*E*_0_|^4^ over the whole nanostructure surface where |*E*_0_| is the mean electric field surrounding the particle ensemble.

## 3 Results

**Fig. 1:**
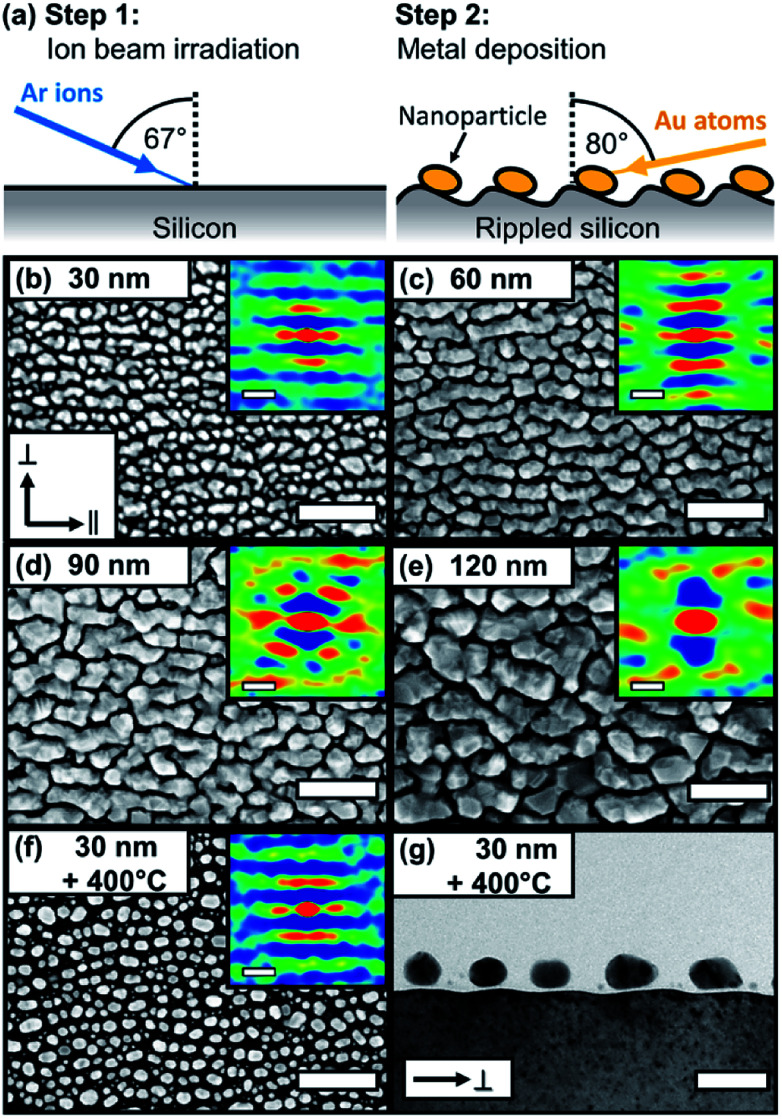
Morphology of self-organized gold nanostructures on rippled templates. (a) Illustration of two-step fabrication routine. (b–f) Top-view SEM images for different gold thicknesses from 30 nm to 120 nm and (f) 30 nm with post-annealing at 400 °C. The insets show the corresponding 2D autocorrelations. (g) Cross-sectional bright-field TEM image of (f). Scale bars (b–f): 200 nm (insets 50 nm), (g) 50 nm.

Fig. 1 shows the morphology development of the plasmonic gold nanostructures during the metal deposition process. As templates, we used ion-beam induced ripple-patterned silicon surfaces with a ripple periodicity of (49.9 ± 2.5) nm (for more details see Methods Section and ESI Fig. S1†). The two-step fabrication routine of SERS substrates with bidirectional plasmon resonances is illustrated in Fig. 1(a). Different morphological steps are observed depending on the gold thickness. Starting with 30 nm, the nanoparticle self-organization with a preferred in-plane orientation along the ripples is clearly observable. By increasing the gold film thickness, the nanoparticles start to elongate and form wire-like nanostructures. With a further increase of the gold thickness, coarser and more complex structures are formed, without the formation of a continuous gold layer. By further increasing the gold thickness, the gold films formed voids reaching a maximum surface coverage of ∼80% (see ESI Fig. S1†). With an additional post-deposition annealing at 400 °C (for 1 hour), we re-shaped the rough and less spherical gold particles into more smooth and spherical ones (f and g). The morphology obtained for annealed gold particles is comparable to the known silver particle chains on rippled templates.^7,8^

Without annealing, rougher particles with less defined shapes are observable.^48,49^ From the 2D autocorrelation and particle analysis, we deduce details on the particle geometry. For gold depositions of 30 nm and 60 nm nominal thicknesses, a clear separation of the structures on the rippled templates is observable. For larger thicknesses of 90 nm and 120 nm this separation is lost, and particle coalescence takes place.

From the 2D autocorrelation, we derived the average center-to-center particle distances for the parallel (∥) and perpendicular (⊥) directions with respect to the pattern axis. The gold nanoparticles follow the periodicity of the template with the maximum particle diameter orientated along (∥ direction) and the minimum diameter across (⊥ direction) the ripples. Table 1 summarizes the particle dimensions for samples with gold thicknesses of 30 nm, 60 nm and for 30 nm Au additionally annealed at 400 °C for 60 minutes. For 90 nm and 120 nm gold deposition thicknesses no isolated structures are formed.

**Table 1:** Summarized dimensions of self-organized gold nanoparticles by annealing

Sample	Min. diameter^a^ (nm)	Max. diameter^a^ (nm)	Particle height (nm)	∥ center-to-center (nm)	⊥ center-to-center (nm)
30 nm	24.4 ± 0.3	36.5 ± 1.4	18.1 ± 2.9	30.3 ± 9.4	46.6 ± 8.7
30 nm, annealed	23.1 ± 0.2	29.8 ± 0.4	17.6 ± 3.5	40.2 ± 12.8	46.9 ± 8.4
60 nm	40.0 ± 0.6	53.1 ± 7.4^b^	—	49.1 ± 8.3	49.1 ± 8.3
	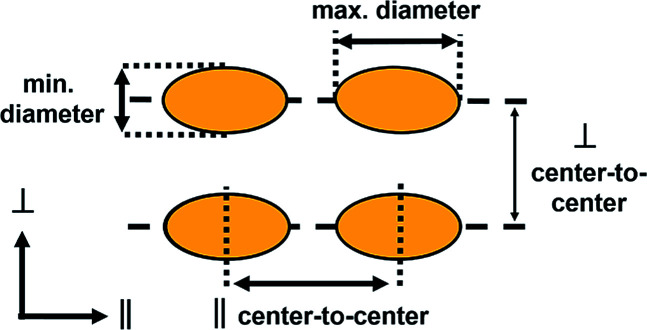				

a Excluding the particles with diameter < 10 nm.

b Excluding the particles grown together over grooves.

**Fig. 2:**
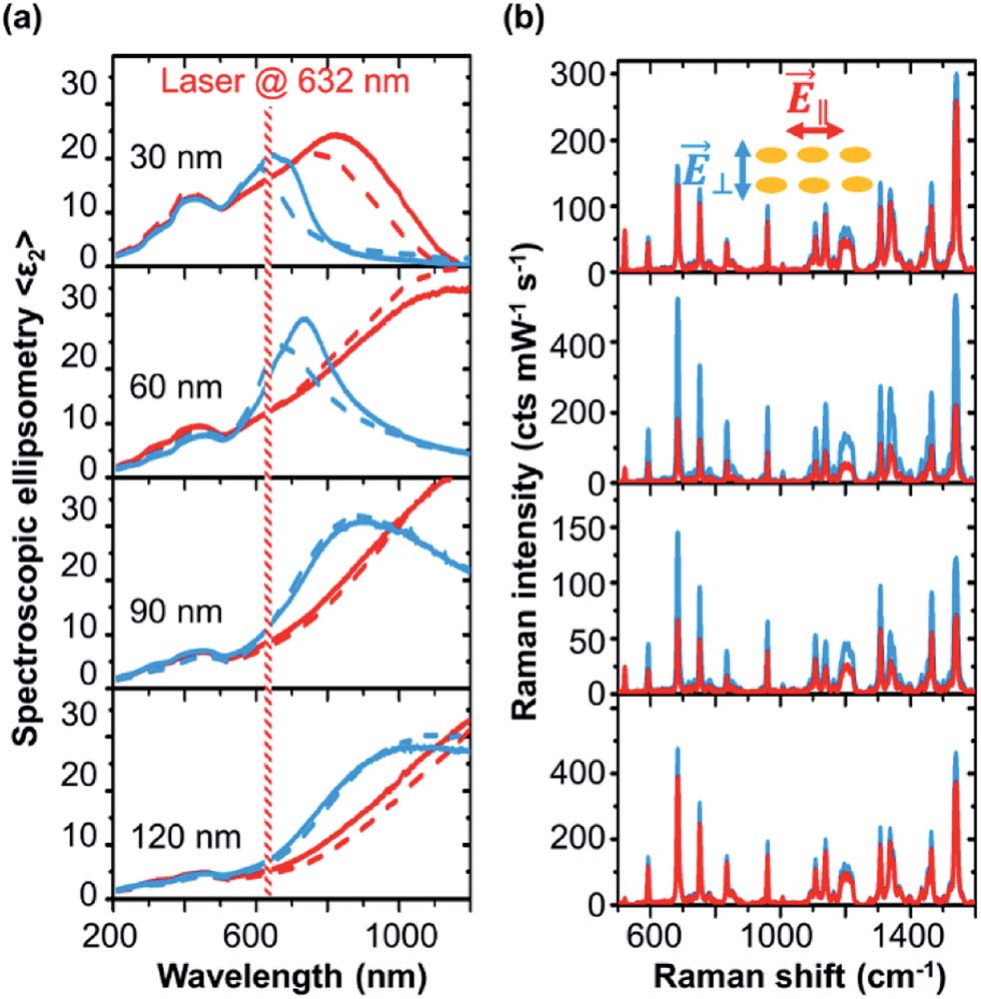
Development of bidirectional LSPR through the gold deposition process. (a) Spectroscopic ellipsometry (SE) of the imaginary part of the effective dielectric function 〈*ε*_2_〉 for polarization parallel (*E⃑*_∥_, red) and perpendicular (*E⃑*_⊥_, blue) to the nanoparticle chains. Solid and dashed lines for spectra with and without deposited CoPc molecules as Raman probe. The Raman excitation laser line of 632.8 nm is indicated. (b) Raman spectra at the same thicknesses corresponding to SE spectra in each column. Raman signal for different laser polarization *E⃑*_∥_ (red) and *E⃑*_⊥_ (blue) are shown.

In Fig. 2, we show the optical properties of the nanostructures for different gold thicknesses. We plot the imaginary part of the effective pseudo dielectric function 〈*ε*_2_〉 measured by spectroscopic ellipsometry. 〈*ε*_2_〉 is associated with the absorption of the Au layer and gives us an indication for the position of the LSPR. The area between 400 to 500 nm is the interband transition of gold, while the plasmon resonance starts in the range of 600 nm and red-shifts for increasing deposition thickness, moving to the mid-infrared range. The LSPR can be excited at different spectral positions for polarizations across (*E⃑*_⊥_) and along (*E⃑*_∥_) the ripple axis. The LSPRs for *E⃑*_⊥_ polarization are observed in the spectral region from ∼600 nm to ∼1000 nm, red-shifting with increasing gold thickness. For *E⃑*_∥_ polarization, the LSPR for 30 nm gold thickness sample starts at ∼800 nm near infrared spectral region and then it red-shifts with increasing gold deposition where the LSPR peak gets broader becoming Drude-like. The results for polarizations across (*E⃑*_⊥_) and along (*E⃑*_∥_) the template show that the LSPRs at different spectral positions can be selectively excited. Evidently, this selectivity is defined by the anisotropic particle shape and coupling along or across the templates. We have probed the plasmonic resonances by Raman spectroscopy with a laser excitation at 632.8 nm, as shown in Fig. 2(b). Using different polarisations Raman signals changes. We analysed the 687 cm^−1^ A_1g_ vibrational mode of CoPc.^50^ The A_1g_ mode has a signal strength of 0.46 intensity counts mW^−1^ s^−1^ on unmodified silicon (see ESI Fig. S2†). As discussed before, the Raman enhancement is given by the intensity ratio of the plasmonic structures with respect to the Raman signal of the molecule on a flat (100) silicon surface used as reference. Enhancement factors from ∼150 up to ∼1200 are observed from the gold nanostructures. SERS enhancement factors (EF) of 10^3^ to 10^4^ are reported for silver nanostructures deposited on rippled silicon templates.^27,28^ Silver nanostructures are well known for their strong plasmon resonances. The benefits of gold nanostructures are the biological and chemical stability and bio-compatibility.

On the first view, our shown SERS effects are comparably low to other SERS substrates and colloidal particle systems.^43,51–55^ In the other hand, SERS enhancement factors are typically given by comparing the ratios of Raman intensities and number of molecules of the SERS substrate and a reference substrate. In the scenario when molecules are diluted in a liquid, the surface affinity of molecules can greatly differ between the reference substrates and the plasmonic nanostructures; this often leads to SERS EF overestimation.^43^ The benefit of the used method in this study is that the SERS enhancement factors can be determined without the need to make assumptions about the molecule concentration.^32^ In this way we can directly probe the plasmonic field enhancement *via* the Raman enhancement factors. This allows the possibility for direct comparison of SERS EFs between different substrates. In comparison to similar self-assembled gold nanostructures our reported SERS EFs are higher or comparable.^4,23–26^ Further, since we can measure and compare the SERS EFs on different substrates, we can directly connect spectroscopic measurements with the Raman field enhancement factors to probe the plasmonic effects on the substrate.

Table 2 summarizes the values for 〈*ε*_2_〉 and SERS enhancement for 632.8 nm excitation wavelength. The highest enhancement is obtained for *E⃑*_⊥_ This means that the signal is dominated by the area between the gold chains since the plasmonic resonance of that direction is excited at this wavelength. For gold thicknesses of 60 nm and 90 nm, a clear anisotropic Raman signal is observed, while for the 30 nm and 120 nm gold thicknesses, the Raman signal anisotropy is less pronounced. These results are in agreement with the absorption due to LSPR deduced from the SE results. We have registered the largest Raman signal amplification for the excitation with 632.8 nm and *E⃑*_⊥_ polarization. The SERS anisotropy EF_⊥_/EF_∥_ is in good agreement with the square of ratio of the pseudo-dielectric function 〈*ε*_2_〉_⊥_^2^/〈*ε*_2_〉_∥_^2^ at this spectral position. The amplitude values of the imaginary part of pseudo dielectric function at the excitation wavelengths are not directly representing the plasmonic excitation strengths. The reason is, that the local plasmonic field enhancement also depends on local geometry of the plasmonic particle. For example, we also observe a strong off-plasmon resonance SERS effect on 120 nm gold structures. This is reasoned by strong electromagnetic field enhancement inside narrow voids (see Fig. 1).

**Table 2:** Summarized Raman enhancement factor EF and pseudo dielectric functions 〈*ε*_2_〉 for *E⃑*_⊥_ and *E⃑*_∥_ polarizations at excitation wavelength at 632.8 nm

Sample	EF_∥_	EF_⊥_	〈*ε*_2_〉_∥_	〈*ε*_2_〉_⊥_	EF_⊥_/EF_∥_	〈*ε*_2_〉_⊥_^2^/〈*ε*_2_〉_∥_^2^
30 nm	289	313	16.2	20.2	1.1	1.1
60 nm	385	1135	12.1	17.8	2.9	3.0
90 nm	144	314	8.5	11.1	2.2	2.0
120 nm	837	1032	5.2	7.0	1.2	1.8

**Fig. 3:**
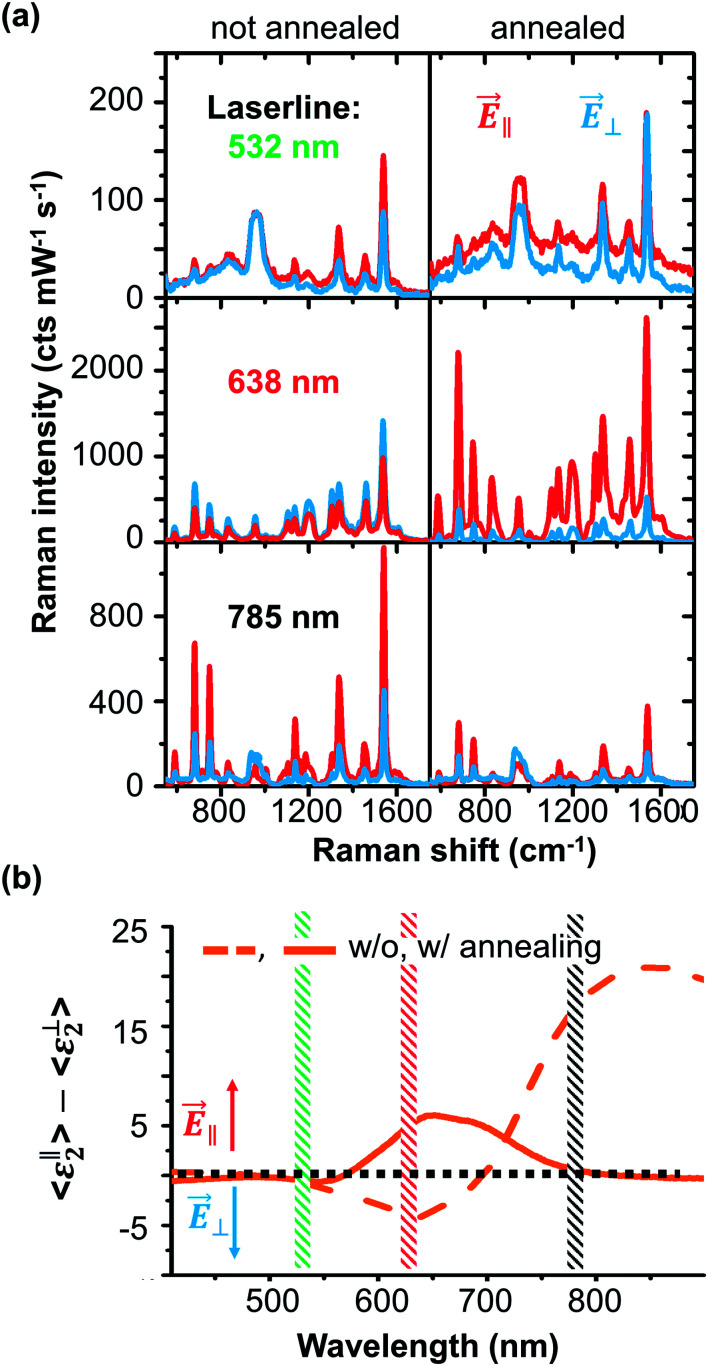
Polarization dependent VIS-NIR Raman responses for 30 nm gold thickness with and without annealing. (a) Raman spectra of 1 nm CoPc probed by three different laser lines (top: 532 nm, middle: 638 nm, and bottom: 785 nm) for parallel (*E⃑*_∥_, red) and perpendicular (*E⃑*_⊥_, blue) polarizations. (b) Difference of the imaginary parts of the pseudo-dielectric function for parallel and perpendicular excitation measured for 30 nm gold thickness without (dashed) and with annealing (solid). Difference below or above zero means that Raman is enhanced for *E⃑*_⊥_ or *E⃑*_∥_ polarization.

Until know we presented the development of LSPR and SERS enhancement for different gold thicknesses deposited on rippled templates. The structures with the most defined geometry and bidirectional LSPRs are the 30 nm gold substrates. By additional post deposition annealing, the gold nanoparticles become more spherical and defined. This reduces the particle center-to-center distances and particle diameter in ∥ direction (see Table 1.). We want to observe the LSPR shift and the effect on SERS by post deposition annealing. In Fig. 3, we compare the anisotropic SERS effect of annealed and non-annealed samples probed by the excitation with three laser lines (532 nm, 638 nm, and 785 nm). With 532 nm, no clear anisotropic effect is observable for both samples. The reason is the off-LSPR excitation, and thus the Raman enhancement is rather low.

For 638 nm excitation, we see a clear anisotropic SERS effect on the annealed sample and a high enhancement for the *E⃑*_∥_ polarization. For the non-annealed sample, a high SERS response for *E⃑*_⊥_ is obtained. This situation reverses for the 785 nm excitation. Here, for the non-annealed sample, a very high SERS enhancement is obtained for the *E⃑*_∥_ direction.

This is a good example for a sample that supports LSPRs excited under two different laser lines which that can be selected by polarization. The 785 nm excitation does not match a LSPR peak of the annealed sample and thus the observed non-resonant SERS enhancement is low and in good agreement with the SE results. The difference of 〈*ε*_2_〉 is shown in Fig. 3(b). Values below zero show an increased LSPR excitation in *E⃑*_⊥_ and above zero in the *E⃑*_∥_ direction. The spectral anisotropy shows that the annealed sample supports an LSPR for *E⃑*_∥_ excitation with a peak around 650 nm. The annealed sample carries two LSPRs matching the 638 nm and 785 nm excitation wavelength, each one individually accessible by controlling the excitation polarization.

**Fig. 4:**
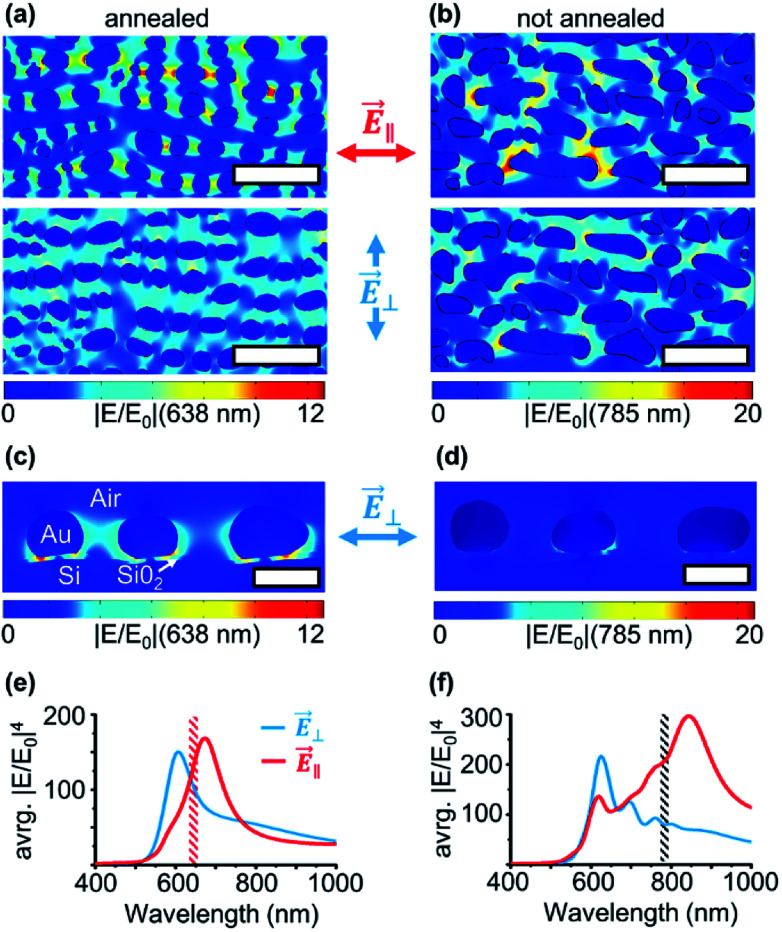
FEM calculation results of the near-field enhancement of electric |*E*/*E*_0_| based on SEM images. The systems modeled correspond to (a) annealed and (b) non-annealed gold samples for two different directions of the incident electric field (top) *E⃑*_∥_ and (bottom) *E⃑*_⊥_. Scale bars: 100 nm. The particle cross-section visualization of the annealed (c) and not annealed (d) sample is shown below (scale 30 nm). Simulated spectra of average SERS enhancement (∝ avrg. |*E*/*E*_0_|^4^) for annealed (e) and not annealed (f) particle geometries for *E⃑*_⊥_ and *E⃑*_‖_ polarizations. Simulated spectra of average SERS enhancement (∝ avrg. |*E*/*E*_0_|^4^) for annealed and not annealed particle geometries for *E⃑*_⊥_ and *E⃑*_∥_ polarizations.

Until now, we experimentally characterized the bidirectional optical properties of the self-organized substrates and probed the signal amplification with SERS. In Fig. 4, we show the spatial distribution of hotspots obtained by finite element method results for the annealed and non-annealed samples. For the annealed sample under 638 nm *E⃑*_∥_ excitation, we can visualize the plasmonic hotspots along the particle chains.

The LSPR coupling between the particles forces the hotspots to follow the particles on the ripple structure, even if the particles are not fully in line with the excitation field. For *E⃑*_⊥_, a lower field enhancement with a weak “far”-coupling across the ripples is visible. In the simulation of the cross-section view in Fig. 4(c), it can be seen that a big portion of the electric field is caged in the native silicon oxide layer between the nanoparticles and the silicon substrate.^56^ Thus for *E⃑*_⊥_ the plasmonic coupling between the nanoparticles is weak and also because of the large separation distance between particles by the ripple structure in the ⊥ direction. Interestingly, even if the particles are tilted because of the ripple pattern, the weak plasmonic interaction follows the shortest distance between the nanoparticles and the dipole-like LSPR field extends into the native oxide layer.

For the non-annealed scenario, the situation becomes more complicated. For the excitation direction *E⃑*_∥_ at 785 nm, strong plasmon hotspots appear especially on particles with an elongated shape. The broad particle size, aspect ratio, and shape distribution result in LSPRs that can be excited in a wide spectral range. The chain structure made by interconnected oval-shaped particles can also contribute to the coupling of hotspots among particles with different shapes.^57^ As we know from the experimental ellipsometry and Raman results, the *E⃑*_⊥_ 785 nm excitation does not match the LSPRs and thus no active hotspots are visible. Interestingly, because of the high aspect ratio of non-annealed particles, a weak plasmonic coupling can also be seen along the particle rows. We summarize in Fig. 4(e and f) the geometry average |*E*/*E*_0_|^4^ spectra for both particle geometries. Since we used a homogenous film of molecules to probe the SERS effect, the amplification of Raman intensity is directly predictable by the EF_SERS_ ∝ |*E*/*E*_0_|^4^ averaged over the whole sample geometry. Our simulated enhancement spectra are in good agreement with the measured 〈*ε*_2_〉 (see ESI Fig. S3†) and anisotropic Raman signal ratios for *E⃑*_⊥_ and *E⃑*_∥_. The calculated enhancements are ∼1–3 times weaker than the experimentally observed signal enhancements (compared to Table 2). This is caused by the fact that most of the signal amplification arises from just a few but very strong hotspots, preferable between very rough particles, therefore the samples cannot be fully represented by the FEM calculations.

## 4 Conclusions

We show a two-step fabrication process to generate gold nanostructures aligned along rippled templates. These structures show localized surface plasmon resonances that can be bidirectionally excited. These LSPRs are tuneable by controlling the fabrication parameters. We demonstrated substrates with LSPRs fitting laser lines from visible to near-infrared spectral regions matching the most conventional excitations used in Raman spectroscopy. We report SERS enhancement of up to ∼1200 times. This SERS signal enhancement is comparatively high for a large-scale self-organized gold films reported in literature.^4,23–26^ Finite element method calculations showed the polarization and wavelength dependent hotspot distributions of gold nanoparticle systems for well-ordered annealed particle films and less well-shaped gold particles deposited at room temperature are observed.

The biocompatible and chemically stable gold nanostructures here reported support strong plasmonic signal enhancement for multiple excitation wavelengths. Because of the time and cost-effective fabrication routine we report, the structures are suitable for mass-fabrication and only a physical vapor deposition system and a low-energy ion beam source are needed. These two requirements are well-established and widely used in research and industry laboratories. We believe that the large-scale self-organized gold nanostructures with bidirectional plasmonic responses have enormous potential for chemical and biological sensing applications where a large number of substrates are needed.

## Conflicts of interest

There are no conflicts to declare.

## Supplementary Material

RA-008-C8RA04031A-s001
