# Supplementary material for: Large-scale self-organized gold nanostructures with bidirectional plasmon resonances for SERS
Source: RSC Adv. 2018 Jun 21;8(40):22569–76. doi: 10.1039/c8ra04031a (PMC9081445; doi:10.1039/c8ra04031a)
Supplement: RA-008-C8RA04031A-s001 [file RA-008-C8RA04031A-s001.pdf]

Please do not adjust margins

Journal Name

ARTICLE

Supplement

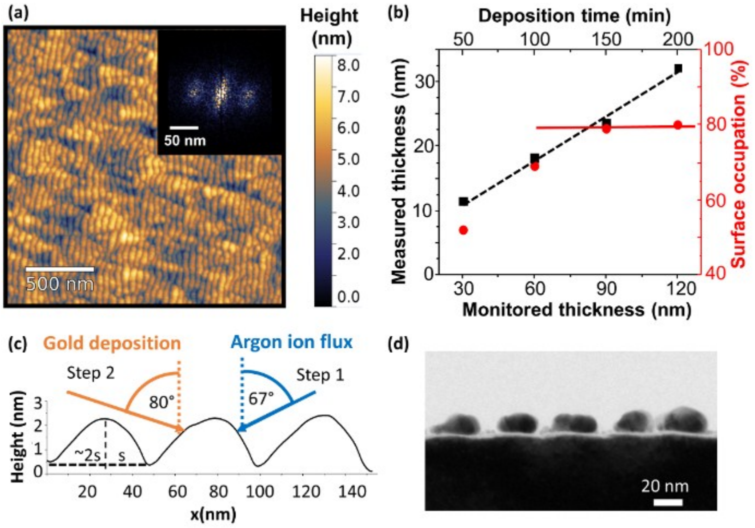

Supplementary figure S1. Morphology of ion induced ripple pattern on silicon surface (a) and topography cross-section (c) below. Inset shows the periodicity of 50 nm of the ripple pattern by 2D Fourier transformation. Incidence angles for ion irradiation and gold deposition are indicated. (b) Rutherford backscattering spectrometry measured film thickness compared to quartz crystal microbalance monitored film thickness. From electron microscopy we extracted the gold surface occupation ratio and imaged the cross-section (d) of gold particles (with 30 nm monitored gold thickness) on the rippled template.

Please do not adjust margins

Please do not adjust margins

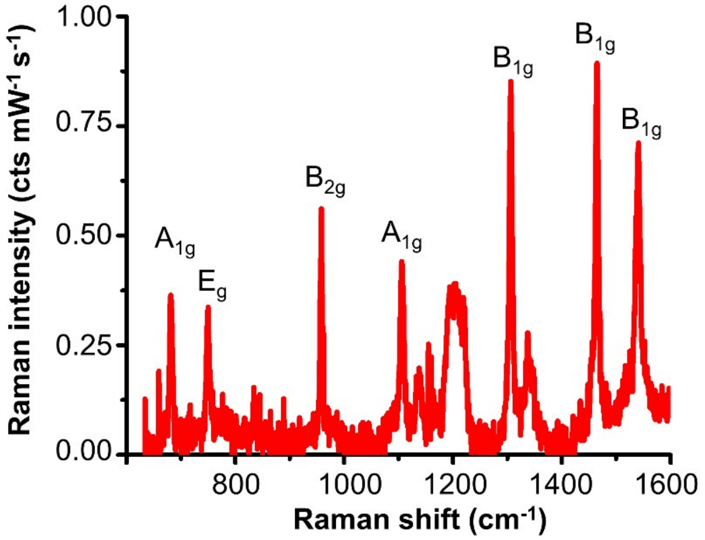

Supplementary figure S2. Raman spectra (HeNe laser excitation 632.8 nm; 1mW; 1s) of CoPc measured on an unmodified (100) silicon surface. Prominent Raman symmetry modes are indicated[1]. The peaks at 1359 cm^{-1} and 1407 cm^{-1} corresponds to $Co-N_4$ asymmetric and symmetric stretching of CoPc.[2]

This journal is © The Royal Society of Chemistry 20xx

Please do not adjust margins

Please do not adjust margins

Journal Name

ARTICLE

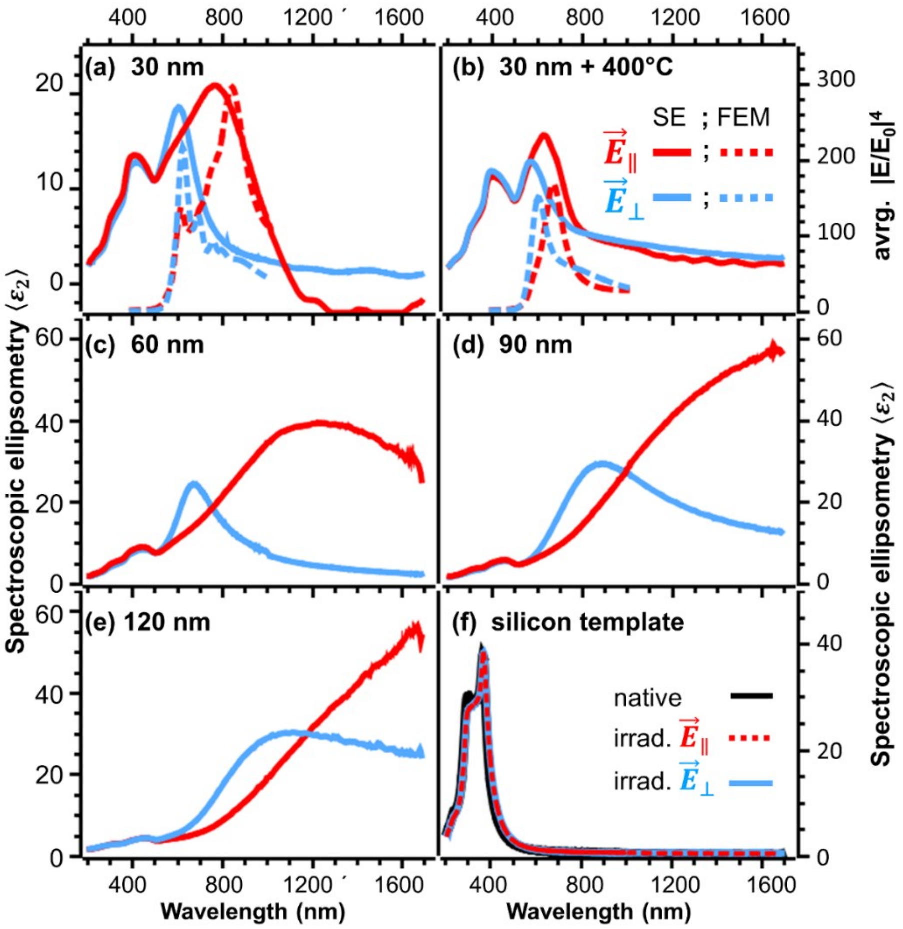

Supplementary figure S3. Overall $\langle\varepsilon_2\rangle$ spectra for different samples measured by spectroscopic ellipsometry (SE). The spectra shown for Gold deposition thicknesses of (a) 30 nm, (b) 30 nm and post-process annealing, (c) 60 nm, (d) 90 nm and (e) 120 nm for polarization parallel ($\vec{E}_\parallel$, red) and perpendicular ($\vec{E}_\perp$,, blue) to the ripple structure. In (a) and (b) corresponding simulated field enhancement spectra avrg. $|E/E_0|^4$ are plotted (FEM, dashed lines). (f) $\langle\varepsilon_2\rangle$ reference spectra for a native silicon substrate (black line) and an ion induced rippled silicon template for polarization parallel ($\vec{E}_\parallel$, dashed red line) and perpendicular ($\vec{E}_\perp$,, solid blue line). During ion beam irradiation absorption peak at ~ 300 nm is reduced, while absorption at 400 nm stays unaffected. The 50 nm rippled silicon substrates does not show a polarization dependence.

This journal is © The Royal Society of Chemistry 20xx

Please do not adjust margins

Please do not adjust margins

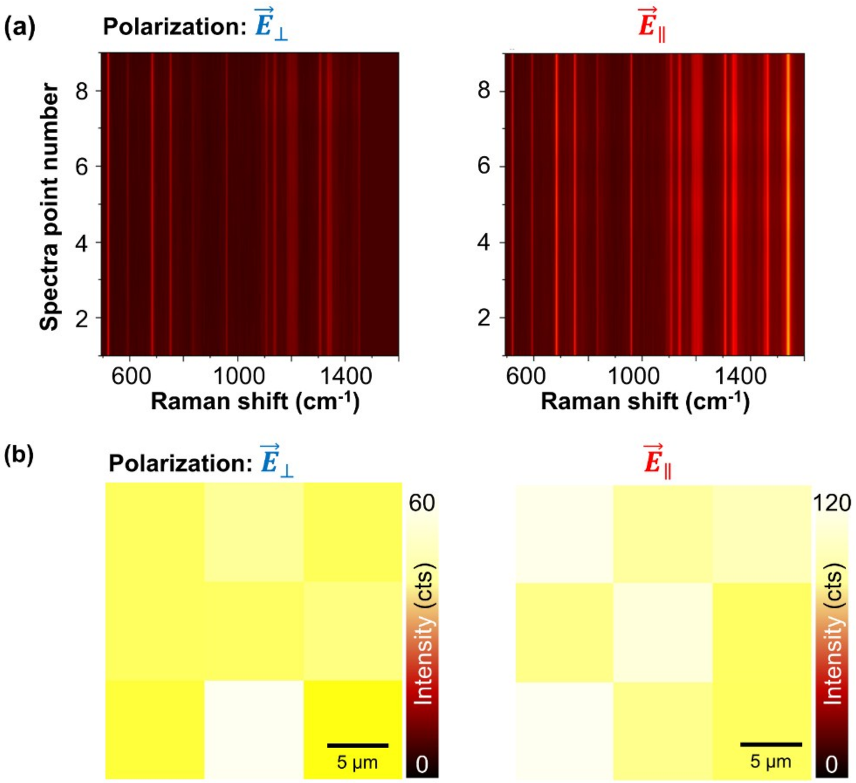

Supplementary figure S4. Raman signal acquisition in a 3x3 grid for CoPc molecules on a self-assembled gold nanostructure under 632.8nm excitation. (a) Raman spot spectra with 1 second collection time for different time points for perpendicular ($\vec{E}_{\perp}$, left) and perpendicular ($\vec{E}_{\parallel}$, right) polarization to the ripple structure. (b) Raman intensity map of CoPc 1542 cm^{-1} mode at 632.8nm.

References

1. Z. Q. Liu, X. X. Zhang, Y. X. Zhang and J. Z. Jiang, *Spectrochimica Acta Part a-Molecular and Biomolecular Spectroscopy*, 2007, **67**, 1232-1246.

2. O. I. Arillo-Flores, M. M. Fadlallah, C. Schuster, U. Eckern and A. H. Romero, *Phys Rev B*, 2013, **87**.

This journal is © The Royal Society of Chemistry 20xx

Please do not adjust margins
